# Ventilating Chambers

**Published:** 1870-12

**Authors:** 


### VENTILATING CHAMBERS.

One-third of our existence is spent in bedrooms, and the difference must be very great in the purification of the blood between breathing a foul and a pure air during this time. Miasm will enter the chambers in the country in warm weather, and burglars in cities in all weathers—and in both city and country there is more or less danger from sudden changes of weather.

In these later days no one is safe anywhere in sleeping with the chamber doors open. Every house-breaker is a would-be-murderer, is always armed for murder, if necessary to protection from discovery or capture. There are two safe methods of ventilating a chamber—let the fire-place remain open; this is not always practicable, sometimes there is no fire-place; and sometimes, too, there are no windows to the chamber, many persons thinking that they can sleep anywhere. Future builders should construct the doors to all rooms, whether chambers or not, in such a way that both at top and bottom a portion of the door three or four inches broad, and two-thirds as long as the door is broad, should be sawed out, and arranged to turn on a pivot at each end, as seen in rail-cars, having a button to fasten it when necessary.

